# Overdose of Quetiapine—A Case Report with QT Prolongation

**DOI:** 10.3390/toxics9120339

**Published:** 2021-12-07

**Authors:** Elisabetta Bertol, Fabio Vaiano, Antonina Argo, Stefania Zerbo, Claudia Trignano, Simone Protani, Donata Favretto

**Affiliations:** 1 Research Unit U.R.I.To.N., University of Firenze, 50134 Firenze, Italy; elisabetta.bertol@unifi.it; 2 Forensic Toxicology Division, Department of Health Sciences, University of Firenze, 50134 Firenze, Italy; fabio.vaiano@unifi.it; 3 Department of Health Promotion, Maternal Child Care and Medical Specialties, University of Palermo, 90151 Palermo, Italy; antonina.argo@gmail.com (A.A.); stefania.zerbo@unipa.it (S.Z.); 4 Department of Biomedical Sciences, University of Sassari, 07100 Sassari, Italy; ctrignano@uniss.it; 5 School of Specialization of Legal Medicine, University Hospital of Padova, Via Falloppio 50, 35121 Padova, Italy; sprotani@gmail.com; 6 Legal Medicine and Toxicology, University Hospital of Padova, Via Falloppio 50, 35121 Padova, Italy

**Keywords:** quetiapine, intoxication, QT-prolongation

## Abstract

Quetiapine is an atypical antipsychotic drug used to treat bipolar disorder, schizophrenia, and major depressive disorder. Although several studies describe the adverse effects of intoxication with Quetiapine, only a few report an extreme overdose without comedications that lead to a life threat. We present a case of a 75-year-old male who tried to attempt suicide by ingesting 28 g of Quetiapine. During the management in the emergency department, both serum and urine samples were collected, allowing a complete pharmacokinetic analysis to be conducted, from the admission to the discharge.

## 1. Introduction

Quetiapine is an atypical antipsychotic drug that belongs to the chemical class of dibenzothiazepine derivatives. It is widely used to treat schizophrenia, acute mania, and depression associated with bipolar disorder, and is adjunctive treatment for major depressive disorder [1,2,3]. Off-label prescriptions of low-dose Quetiapine are not recommended [4].

Analogously to other atypical antipsychotics, Quetiapine has fewer extrapyramidal side effects than typical antipsychotics, which may contribute to better patient compliance; it can be used for both positive and negative symptoms and does not induce prolactin elevation [3,5,6,7].

Quetiapine interacts as an antagonist with a wide range of neurotransmitter receptors in the brain. It has been proposed that its antipsychotic activity is mediated through a combination of dopamine type2 (D2) and serotonin type2 (5-HT2) antagonism. It also has a high affinity for α1-adrenergic and histamine receptors but a lower affinity for muscarinic receptors. Nevertheless, the exact mechanism of action of Quetiapine, as with other antipsychotic drugs, is still unknown [5].

Quetiapine is rapidly absorbed from the gastrointestinal tract, with peak plasma concentrations reached within two hours after oral administration [6]. It is 83% plasma protein bound [8], widely distributed throughout the body, with a volume of distribution of 8–10 L/kg [6], and extensively metabolized in the liver by sulfoxidation, carboxylic acid formation on the ethoxyethanol side chain, and 7-hydroxylation. At least 20 metabolites have been detected, but only 7-hydroxyquetiapine has significant pharmacological activity [9]. Quetiapine in the serum has a half-life of about six hours. Steady-state concentrations are achieved within two days of dosing, which usually starts with a dose of 25 mg b.i.d., titrated with increments of 25–50 mg/day b.i.d., as tolerated, to a target daily dose in adults from 150 to 750 mg (with further dosage adjustments depending on the clinical response and tolerance) [5,6,8,10,11]. The effective dose is 200–750 mg daily with an average therapeutic concentration of 0.4 mg/L. Linear pharmacokinetics are observed [9].

Reported signs and symptoms of an overdose are attributable to an enhancement of the known pharmacological effects of the drug; the antagonism of histamine H1-receptors explains sedation and somnolence. Orthostatic dysregulation, hypotension, and tachycardia are associated with an antagonistic effect on α1-adrenergic receptors. Quetiapine has also been reported to have an antagonistic effect on M1-muscarinic receptors resulting in anticholinergic-mediated tachycardia [5,6,10,12]. Overdose can also lead to QT prolongation, seizures, status epilepticus, rhabdomyolysis, respiratory depression, urinary retention, confusion, delirium or agitation, coma, and death. Patients with pre-existing severe cardiovascular disease may be at increased risk of developing overdose effects.

There is no specific antidote for Quetiapine. In cases with more severe manifestations, the possibility of multiple drug involvement should be considered, and intensive care procedures are recommended, including establishing and maintaining a patent airway to support adequate oxygenation and ventilation, monitoring, and support of the cardiovascular function.

According to the published literature, patients with delirium and agitation and an evident anticholinergic syndrome can be treated with 0.5–1 mg of physostigmine [13,14] (under continuous ECG monitoring). Using this drug is not recommended as a standard treatment due to the potential adverse effect of physostigmine on the conductance of the heart. Physostigmine can be used in the absence of ECG aberrations; therefore, it must not be used in case of arrhythmias, any degree of heart block, or enlargement of the QRS complex [14,15]. Although prevention of absorption in overdose has not been evaluated, gastric lavage may be considered in cases of severe intoxication, possibly within one hour of ingestion. The administration of activated charcoal should also be considered. In cases of Quetiapine overdose, refractory hypotension should be treated with appropriate measures, such as intravenous fluids or sympathomimetic agents. Epinephrine and dopamine should be avoided, as beta stimulation may worsen hypotension during the onset of the quetiapine-induced alpha blockade. Careful medical supervision and appropriate monitoring must be ensured until the patient is cured [14].

The case we present here deals with an extreme overdose of Quetiapine in which we could evidence the side effects and pharmacokinetics of that drug.

## 2. Case Report

We report on a 75-year-old retired male with a history of depression and bipolar disorder characterized by severe to moderate maniacal periods, treated with 200 mg of Quetiapine per day. He was living by himself, occasionally helped by a housekeeper, when he was found unconscious on the floor, in a semi-deep coma state, by his son.

Six packages of the drug “Quetiapine Teva”, each containing 60 pills of 100 mg of Quetiapine, were found in the patient’s home. Overall, 280 pills were missing, tantamount to 28 g of Quetiapine, which the subject declared to have ingested for approximately one hour in an attempt to commit suicide. The patient claimed he intended to ingest all his stock, but he fainted before fulfilling his intention.

The emergency medical service found the patient comatose and transported him to the emergency room where he suddenly presented chest pain followed by a cardiac arrest. Cardiopulmonary resuscitation was attempted with an automatic defibrillator. The ECG tracing detected an increase in the QT-interval, normalized after resuscitation maneuvers. To prevent further absorption of Quetiapine, gastric content was aspired, followed by gastric lavages, and activated charcoal was administered enterically.

Serum and urine samples were collected on arrival at the emergency department and in the following three days (eight serum samples and seven urine samples in total). Quetiapine concentrations were determined by HPLC-MS/MS. Biological specimens (200 µL) were added to 0.5 mL of NaOH 2 N, 10 µL of internal standard (halazepam, 50 ng/µL), and liquid–liquid extracted (LLE) with 5.0 mL of a 9:1 (v:v) dichloromethane/isopropanol mixture. After centrifugation at 4000 rpm for 5 min, the lower organic layer was transferred into a tube, dried under a gentle stream of nitrogen at 40 °C, and the residue dissolved in 100 µL of LC–MS methanol. An aliquot of 3 µL was injected into a HPLC Agilent 1290 Infinity system (Agilent Technologies, Palo Alto, CA, USA) interfaced with an Agilent 6460 triple quadrupole LC-MS/MS (Agilent Technologies) and an electrospray (ESI) ion source. The column used was a Zorbax SB-C18 Rapid Resolution HT (2.1 × 50 mm, 1.8 µm, Agilent Technologies), heated at 30 °C. The mobile phase initially consisted of 5 mM aqueous acid formic (A) and acetonitrile (B) 90:10. Gradient elution was carried out at a flow rate of 0.4 mL/min. The positive ESI conditions were: gas temperature 325 °C; gas flow rate 10 L/min; nebulizer 20 psi; capillary voltage 4000 V. Multiple reaction monitoring (MRM) analysis was performed in the positive mode for the following transitions: 384:253, 221 *m*/*z* for quetiapine; 353:241, 222 *m*/*z* for halazepam. Data acquisition and elaboration were performed by the Agilent Mass Hunter Workstation Software (Agilent Technologies, Palo Alto, CA, USA). The lower limits of quantifications for serum and urine were 0.5 and 0.1 ng/mL, respectively. The results are reported in Table 1.

**Figure 1:**
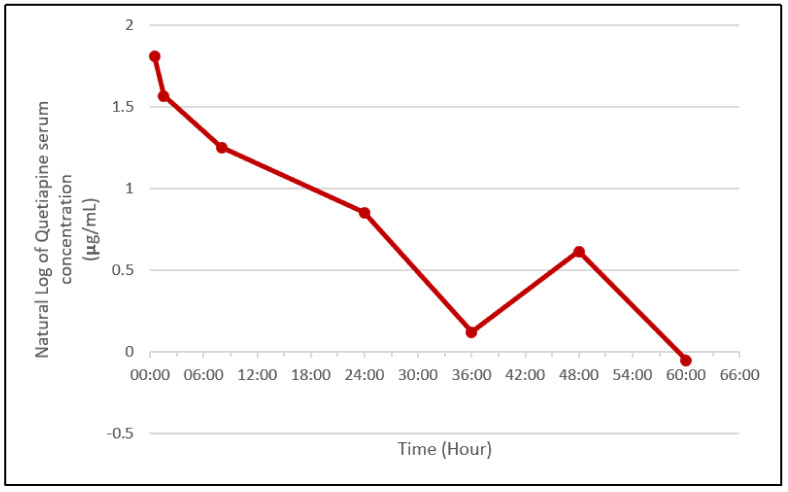
Serum time-related natural log of Quetiapine concentration.

**Figure 2:**
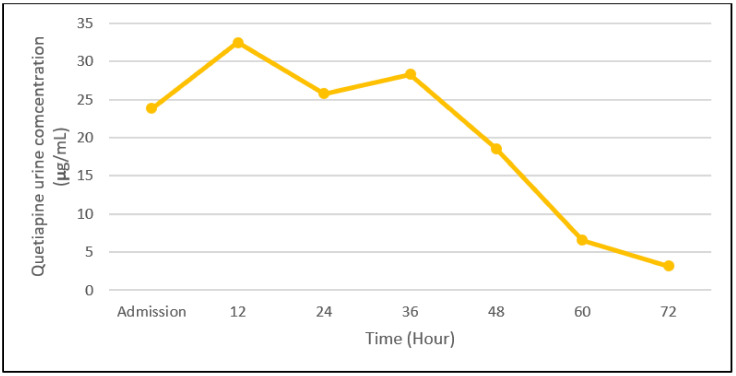
Urine time-related concentration of Quetiapine.

Figure 1 and Figure 2 summarize the Quetiapine concentration trend, piecing together the collected pharmacokinetic data.

Moreover, Quetiapine concentrations were also determined in the gastric aspirate liquid and the fluid of the gastric lavages (Table 2).

Therefore, at the moment of admission about 3.465 g of Quetiapine were in the gastric content, not yet absorbed, suggesting that the subject had absorbed about 24.5 g/28 g of the drug. It must be highlighted that for Quetiapine activated charcoal is a detoxifying practice because reduction in resorption by 35% with activated charcoal administration 0.5 to 6 h after ingestion is described [16].

## 3. Discussion

This case report represents one of the few cases described in the literature of an extremely severe Quetiapine intoxication. Among the possible adverse effects reported, the subject developed coma and increased QT-interval leading to a cardiac arrest, resolved only due to the timely medical intervention. Successful treatment included oxygenation, ventilation, hemodynamic support, gastric lavage, and administration of activated charcoal under constant electrocardiographic monitoring. Therefore, although the literature reports that Quetiapine is relatively safe in overdose [17], the case report we present highlights Quetiapine lethality when taken alone and at high, clearly over therapeutic, dosages.

Quetiapine half-life in serum is reported in the literature to be approximately five to seven hours [9,11] in clinical studies, at therapeutic dosages; from the data we collected at the admission and 8 h later, this half-life was confirmed, even for the extremely high dosage of about 24 g. However, the terminal half-life was much higher, because the level determined at 8 h required an additional 24 h to halve. A similar observation was made by Nudelman [17], who proposed two possible explanations may have been responsible: nonlinear or saturation kinetics development when Quetiapine reaches higher plasma levels, or CYP3A4 inhibition by the concurrent overdose of fluoxetine. As there was no co-intake of fluoxetine, the case we report allows us to exclude the second hypothesis. Another possibility was proposed by Pollak [18]: new agents that are active at low concentrations, such as Quetiapine, have their terminal elimination half-lives easily underestimated. This reflects the fact that pharmacokinetic studies for product registration are carried out in healthy volunteers with single oral doses of the medication. Single doses of high-potency agents with long half-lives do not generate serum concentrations that remain above the limit of detection of the laboratory assay long enough to document the actual terminal elimination half-life.

The increase in Quetiapine concentration detected between 36 and 48 h post-admission is consistent with reports in previous works [9] that noted an increase in quetiapine concentration between 24 and 37 h, probably attributable to hepatic redistribution.

Toxic serum-plasma concentrations of Quetiapine, previously described in the literature, are between 1 and 1.8 mg/mL, whereas comatose/fatal concentrations are between 1.9 and 12.7 mg/L [18,19]. Table 3 summarizes the findings of Pollak [18], Harmon [19], and the current study regarding Quetiapine’s toxicity.

In the case we reported, the maximum serum concentration of Quetiapine was 6.45 mg/L evaluated on admission. Nevertheless, it should be considered that the first assessment of the serum concentration of Quetiapine took place, according to the declaration of the patient, about 4 h after intoxication. Therefore, assuming a half-life of 6 h, at least in the initial post-intake phases, serum concentrations of Quetiapine may have reached a serum peak of approximately 10 mg/L. Moreover, it should be noted that many cases of Quetiapine overdoses, in which even higher serum concentrations of the drug were described, did not cause the onset of malignant arrhythmias. This highlights that the previous conditions of the subjects are fundamental in establishing the toxic and lethal doses of this substance.

It is certain in the present case that QT prolongation had occurred, and heart attack would have been fatal if resuscitation was not performed.

## 4. Conclusions

The present case, describing the consequences of a voluntary, suicidal overdose of Quetiapine alone (28 g), highlights the adverse effects and lethality of Quetiapine when cardiopulmonary resuscitation is not available. The observed effects were explained by the known pharmacology data of Quetiapine, for which the most severe side effect is the onset of arrhythmia, coma, and respiratory depression. It is clear that management of Quetiapine intoxication should include intensive care procedures and ECG monitoring, in conjunction with measures to prevent absorption from gastric content.

The pharmacokinetic data we reported are in agreement with the literature, except for the terminal half-life, which is also reflected in previous publications on Quetiapine intoxication cases. Further investigations are needed to understand if the half-life evaluated in the clinical studies during product registration was underestimated or non-linear pharmacokinetics occurs at high doses.

## Figures and Tables

**Table 1:** Serum and urine time-related concentration of Quetiapine after the assumed ingestion of 28 g of Quetiapine in a 75-year-old male patient.

	Admission	30 min aa	90 min aa	8 h aa	12 h aa	24 h aa	36 h aa	48 h aa	60 h aa	72 h aa
**Urine** (µg/mL)	23.83				32.50	25.80	28.32	18.56	6.56	3.15
**Serum** (µg/mL)	6.45	6.12	4.80	3.50		2.35	1.13	1.85	0.95	

aa = after admission

**Table 2:** Gastric aspirate and liquid of gastric lavage concentration of Quetiapine.

Quetiapine	Gastric Aspirate (200 mL)	I Gastric Lavage (200 mL)	II Gastric Lavage (200 mL)	Following Gastric Lavages
**concentration** **(quantity)**	16,177 µg/mL (3235 mg)	1100 µg/mL (220 mg)	50 µg/mL (10 mg)	Undetected

**Table 3:** Quetiapine’s overdose symptoms described by Pollak [18] and Harmon [19], and of our case.

REF	Age	Ingested amount	Time-related symptoms after the overdose				
			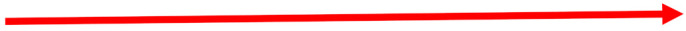				
			3 h	5h	9 h	24 h	48 h
[18]	40	3000 mg	Hypotension (90–55 mm Hg); Tachycardia (120 bpm) [PR and QT intervals at upper limit of normal]; GCS 14/15.	GCS 8/15	GSC 10/15	Tachicardia	Normal conditions
[19]	26	2000 mg	Tachycardia with a normal QRS complex	Loss of consciousness	GSC 6/15	Tachicardia	Normal conditions
This case	75	2800 mg	Loss of consciousness	Comatose	QT prolongation, chest pain and cardiac arrest	QT prolungation	Normal conditions

## Data Availability

Department of Health Sciences, Forensic Toxicology Division, University of Firenze, Firenze, Italy.

## References

[1] Komossa K., Depping A.M., Gaudchau A., Kissling W., Leucht S. (2010). Second-generation antipsychotics for major depressive disorder and dysthymia. Cochrane Database Syst. Rev..

[2] The American Society of Health-System Pharmacists Quetiapine Fumarate. www.drugs.com/monograph/quetiapine.html.

[3] Arvanitis L.A., Miller B.G. (1997). Multiple fixed doses of “Seroquel” (quetiapine) in patients with acute exacerbation of schizophrenia: A comparison with haloperidol and placebo. The Seroquel Trial 13 Study Group. Biol. Psychiatry.

[4] Government Executive Media Group. Oregon State Drug Use Evaluation: Low Dose Quetiapine (Seroquel/Seroquel XR). web.archive.org/web/20160422184014/http://www.govexec.com/pdfs/013111bb1a.pdf.

[5] Müller C., Reuter H., Dohmen C. (2009). Intoxication after extreme oral overdose of quetiapine to attempt suicide: Pharmacological concerns of side effects. Case Rep. Med..

[6] Langman L.J., Kaliciak H.A., Carlyle S. (2004). Fatal overdoses associated with quetiapine. J. Anal. Toxicol..

[7] Nasrallah H.A., Tandon R. (2002). Efficacy, safety, and tolerability of quetiapine in patients with schizophrenia. J. Clin. Psychiatry.

[8] Anderson D.T., Fritz K.L. (2000). Quetiapine (Seroquel) concentrations in seven postmortem cases. J. Anal. Toxicol..

[9] Moffat A.C., Osselton M.D., Widdop B., Watts J. (2011). Clarke’s Analysis of Drugs and Poisons in Pharmaceuticals, Body Fluids and Postmortem Material.

[10] Hasselstrøm J., Linnet K. (2004). Quetiapine serum concentrations in psychiatric patients: The influence of comedication. Ther. Drug Monit..

[11] DeVane C.L., Nemeroff C.B. (2001). Clinical pharmacokinetics of quetiapine: An atypical antipsychotic. Clin. Pharmacokinet..

[12] Leucht S., Cipriani A., Spineli L., Mavridis D., Orey D., Richter F., Samara M., Barbui C., Engel R.R., Geddes J.R. (2013). Comparative efficacy and tolerability of 15 antipsychotic drugs in schizophrenia: A multiple-treatments meta-analysis. Lancet.

[13] Dawson A.H., Buckley N.A. (2016). Pharmacological management of anticholinergic delirium—Theory, evidence and practice. Br. J. Clin. Pharmacol..

[14] Riassunto delle Caratteristiche del Prodotto Document Made Available by A.I.F.A. on 09 July 2021. farmaci.agenziafarmaco.gov.it/aifa/servlet/PdfDownloadServlet?pdfFileName=footer_002322_041024_RCP.pdf&retry=0&sys=m0b1l3.

[15] Arens A.M., Kearney T. (2019). Adverse effects of physostigmine. J. Med. Toxicol..

[16] Isbister G.K., Friberg L.E., Hackett L.P., Duffull S.B. (2007). Pharmacokinetics of quetiapine in overdose and the effect of activated charcoal. Clin. Pharmacol. Ther..

[17] Nudelman E., Vinuela L.M., Cohen C.I. (1998). Safety in overdose of quetiapine: A case report. J. Clin. Psychiatry.

[18] Pollak P.T., Zbuk K. (2000). Quetiapine fumarate overdose: Clinical and pharmacokinetic lessons from extreme conditions. Clin. Pharmacol. Ther..

[19] Harmon T.J., Benitez J.G., Krenzelok E.P., Cortes-Belen E. (1998). Loss of consciousness from acute quetiapine overdosage. J. Toxicol. Clin. Toxicol..

